# Effects of metabolic syndrome on pulmonary infection in pediatric bronchial asthma: a narrative review

**DOI:** 10.3389/fped.2026.1770376

**Published:** 2026-06-23

**Authors:** Li He, Yang Ye

**Affiliations:** Department of General Pediatrics, Longquanyi District Maternal and Child Health Hospital, Chengdu, Sichuan Province, China

**Keywords:** bronchial asthma, children, metabolic syndrome, obesity, pulmonary infection

## Abstract

Bronchial asthma is a heterogeneous disease characterized by chronic airway inflammation and airway hyperresponsiveness. It is the most common chronic airway inflammatory disease in children and severely affects their physical and mental health. The exacerbation of asthma in children often involves interactions among environmental triggers, the airway microbiome, and the innate immune response. Studies have confirmed that asthma in children is closely associated with lung infections. On the one hand, asthma in children increases the likelihood of lung infections; on the other hand, lung infections can significantly increase the likelihood of acute asthma attacks in children. Metabolic syndrome in children and adolescents is considered a risk factor for chronic diseases such as diabetes and cardiovascular and cerebrovascular diseases. Recent studies have shown that metabolic abnormalities in children are significantly associated with pulmonary infections and asthma in children. This review aims to review and analyze the specific effects of metabolic abnormalities on pulmonary infections and asthma exacerbations in children with asthma. Metabolic abnormalities in children cause chronic inflammation and alterations in the intestinal flora, which affect lung function, promote lung infection, and aggravate bronchial asthma in children.

## 1 Introduction

Chronic respiratory disease is a type of illness that has a long-term impact on the functions of the respiratory tract or lungs, with common types including bronchial asthma, chronic obstructive pulmonary disease (COPD), bronchiectasis, and interstitial lung disease (1, 2). Asthma is a prevalent chronic respiratory disease affecting more than a quarter of a billion people worldwide and is the most common chronic condition among children (3). The incidence of asthma in children is significantly greater than that of adult asthma. Globally, approximately one in ten children will suffer from asthma, with the prevalence peaking between the ages of 5 and 9. Furthermore, the prevalence rate among males aged 0–14 years is higher than that among females (4–6). Although recent studies have reported a decrease in the prevalence of asthma compared with that in 1990, the absolute number of children with asthma continues to increase as the global population expands, and the burden of asthma is projected to further increase (7). Clinically, asthma is characterized by chronic eosinophilic airway inflammation, fluctuating airflow limitations, and symptoms, including wheezing, shortness of breath, chest tightness, and coughing (3, 7).

In accordance with the 2025 Global Initiative for Asthma (GINA) treatment guidelines, the management of asthma in children should incorporate inhaled corticosteroids (ICSs). Given that children are in a developmental stage, their treatment regimen (for those aged 6–11 years) differs from that of adults. A stepwise treatment approach is adopted as follows: 1) short-acting beta-2 agonist (SABA) combined with low-dose ICS on an as-needed basis; 2) daily low-dose ICS plus SABA; 3) low-dose ICS-long-acting beta-2 agonist (LABA)/medium-dose ICS/very low-dose ICS-LABA/low-dose ICS-maintenance-and-reliever therapy (MART), with referrals if uncontrolled; and 4) a specialist assessment, with the addition of a long-acting muscarinic antagonist (LAMA)/biologics (8, 9). Although ICSs demonstrate significant efficacy in bronchial asthma by reducing inflammation and preventing structural changes in the airways, approximately 30%–50% of adults diagnosed with asthma and treated with ICSs do not achieve well-controlled asthma, even when combined with a LABA (10).

**Figure 1:**
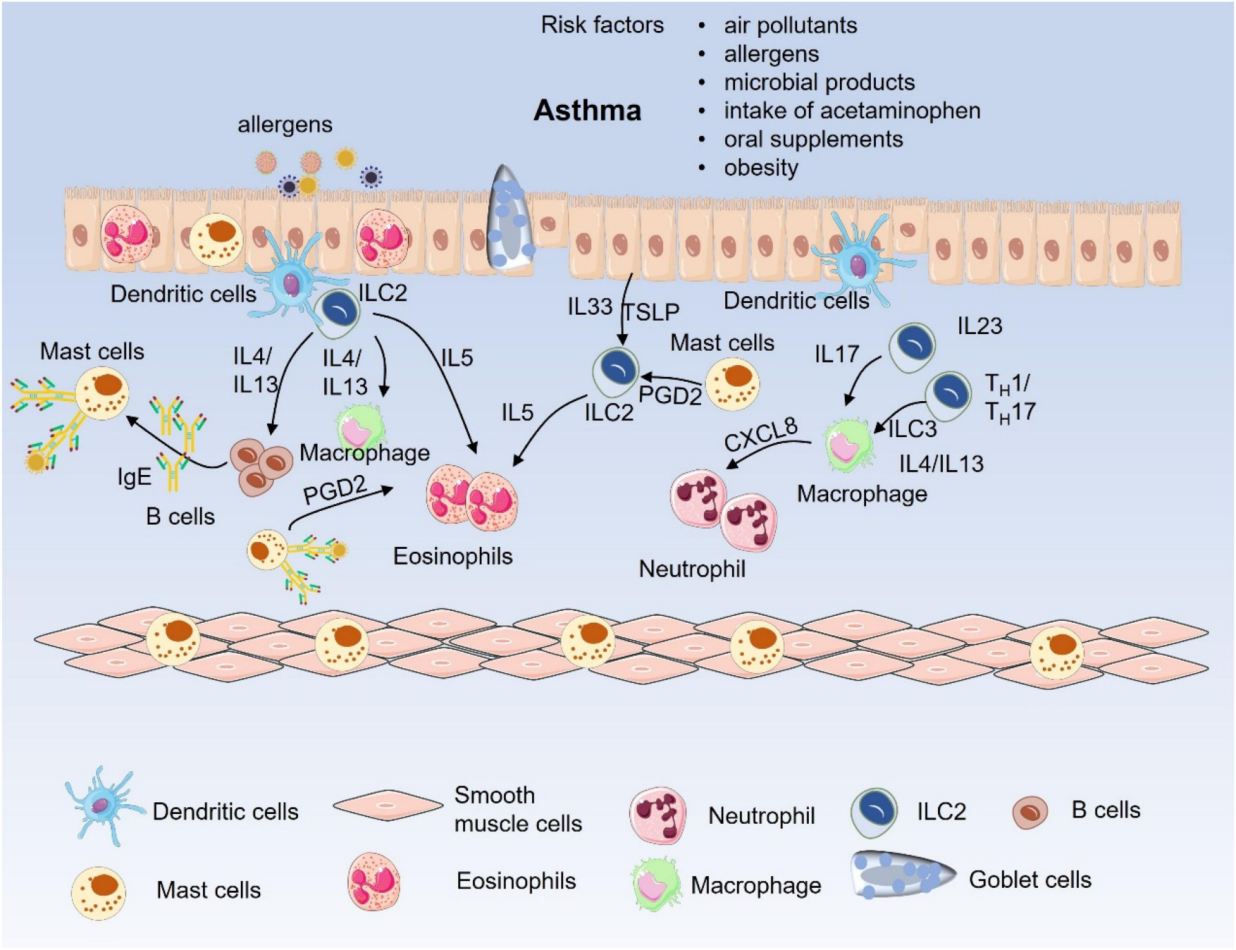
Potential mechanisms of bronchial asthma. The main pathogenic mechanisms of asthma include eosinophilic inflammation, characterized by high eosinophil infiltration, epithelial damage, reticular basement membrane thickening, and airway smooth muscle mass. NNE inflammation is mediated by neutrophilic type 1 and type 17 and paucigranulocytic inflammation. Innate lymphoid cells become activated by PGD2, IL-33, and TSLP, which are released following damage to epithelial cells caused by pollutants or microbes.

Pediatric bronchial asthma is defined as chronic airway inflammation, airway hyperreactivity (AHR), and intermittent (sometimes persistent) airflow obstruction. The etiology of asthma is multifactorial and can influence its severity, including environmental factors (e.g., air pollution and chemical irritants), genetic predispositions, autoimmune responses to allergens (e.g., pollen), and comorbidities (e.g., pulmonary infections or metabolic disorders) (11). Pulmonary infections can exacerbate asthma symptoms. Over 90% of pediatric asthma exacerbations are caused by viruses, such as *rhinoviruses* (RVs) and *human respiratory syncytial virus* (hRSV) (12, 13). The pathogenic mechanisms involved in asthma are heterogeneous in children and adults. Approximately one-half of adults with asthma exhibit eosinophilic inflammation, characterized by high eosinophil infiltration, epithelial damage, reticular basement membrane thickening, and airway smooth muscle mass (14). In nonallergic eosinophilic asthma, innate lymphoid cells respond to prostaglandin D2 (PGD2) and epithelial alarmins—interleukin-33 (IL-33), IL-25, and thymic stromal lymphopoietin, which are released following epithelial injury caused by pollutants or microbes (15, 16). In addition, group 2 innate lymphoid cells (ILC2s), which are key controllers of type 2 inflammation, are involved in the pathogenesis of asthma through the activation and production of type 2 cytokines (17) (Figure 1).

Metabolic disorders refer to pathological conditions characterized by abnormal metabolism of proteins/amino acids, fats, and carbohydrates, including phenylketonuria, dyslipidemia and hyperglycemia. These conditions can lead to obesity, diabetes, metabolic syndrome (MetS), nonalcoholic fatty liver disease (NAFLD), gut microbiota dysbiosis, or hyperinsulinemia (18–20). Metabolic disorders trigger the remodeling of metabolic pathways and chronic inflammation (21). Within a metabolic microenvironment dominated by hyperglycemia and dyslipidemia, immune cells can be activated and produce inflammatory mediators. For example, effector T cells exhibit increased release of proinflammatory factors, while regulatory T-cell function is impaired. Macrophages are transformed into proinflammatory M1-type macrophages, promoting tissue inflammation and insulin resistance (22). Metabolic abnormalities drive the body to generate persistent inflammatory responses, thereby exacerbating inflammation during pneumonia (23). Metabolic abnormalities are also associated with asthma. For example, obesity is significantly associated with the development and severity of asthma in children and adults, and weight loss is associated with improved asthma (24). Studies have reported that metabolic disorder-induced obesity may impair lung function in children with asthma after coronavirus disease 2019 (COVID-19) infection (25). Metabolic disturbances also affect hormone secretion. For example, leptin is an adipokine secreted by adipocytes and enhances energy consumption. Elevated leptin enhances the production of Eotaxin (CCL11), monocyte chemoattractant protein 1 (MCP-1, CCL2), C-X-C motif chemokine ligand 8 (CXCL8), CXCL10, and IL-6, thus contributing to bronchial hyperreactivity and worsening of asthma in children with obesity (26). Compared with obesity, hyperinsulinemia associated with insulin resistance is a stronger risk factor for asthma. Insulin resistance induces airway remodeling, increases bronchial reactivity, and promotes the production of proinflammatory mediators from adipose tissue (27).

Given the close associations among asthma, pulmonary infections, and metabolic abnormalities, this review aims to examine the specific effects of metabolic abnormalities on pulmonary infections and asthma exacerbations in children. By elucidating how metabolic abnormalities in children lead to chronic inflammation, increased secretion of proinflammatory mediators, and negative effects on lung function, we explore how these mechanisms contribute to the development of pulmonary infections and the worsening of bronchial asthma in children.

## 2 Literature search method

### 2.1 Literature retrieval

This study adopted a comprehensive retrieval strategy and conducted systematic searches of English literature through databases such as PubMed and Web of Science. Additionally, Chinese literature resources, including the China National Knowledge Infrastructure (CNKI) and Wanfang Database, were supplemented for retrieval. The search scope was updated to March 31, 2026.

### 2.2 Search terms

The relevant literature was manually screened on the basis of keywords related to asthma or bronchial asthma, metabolic disorders (metabolic abnormalities, MetS, insulin resistance, OR obesity), pulmonary infections (pneumonia OR respiratory infections), and pediatric (children, pediatrics, childhood, OR adolescents).

### 2.3 Inclusion criteria

We included all types of study designs (randomized controlled trials, cohort studies, case‒control studies, cross-sectional studies, etc.), as well as systematic reviews, meta-analyses, and narrative reviews. Two investigators independently screened abstracts available in the literature and reviewed all potential studies included in the reviews. Any discrepancies were resolved through discussion or, when necessary, by a third reviewer. Manual backward citation was performed on the basis of references from the included studies to supplement additional relevant research.

## 3 The association between pulmonary infection and pediatric bronchial asthma

Pediatric pulmonary infections play important roles in the exacerbation of pediatric asthma and reduce the quality of life of patients. Dysbiosis of the pulmonary microbiome may be a key factor in the onset and progression of asthma, triggering persistent inflammatory responses and impairing immune function (28). The etiology of pulmonary infection is complex and involves mainly pathogenic microorganism infection and various physicochemical factors (such as chemical substances). Pathogenic microorganism infections can be roughly divided into viral infections (approximately 90% of cases) and other infections (such as bacterial infections, fungal infections, and mycoplasma infections) (12, 13, 28).

### 3.1 Pediatric asthma complicated with bacterial pneumonia

In cases of bacterial pneumonia, common pathogenic microorganisms include *Streptococcus pneumoniae* (Spn), *Staphylococcus aureus* (SA), and *Haemophilus influenzae* (Hi). Infants colonized with Spn exhibit an increased risk of their first wheezing episode and are more prone to progression to persistent wheezing or asthma, along with a significantly elevated risk of asthma-related hospitalization (29). Compared with children without asthma, children with asthma face a greater risk of invasive pneumococcal disease and pneumonia (30). Even after being vaccinated with the 7-valent pneumococcal conjugate vaccine, these children demonstrate more severe disease progression than nonasthmatic and age-matched controls do (30). Children with asthma treated with ICS have a higher risk of pneumococcal colonization than controls do (31). Early nasopharyngeal colonization with Spn is closely related to eosinophilia, elevated serum total IgE levels, and reversible airway changes, all of which increase the risk of asthma in children. This may be associated with the binding of phosphatidylcholine in the Spn cell wall to platelet-activating factor receptor (PAFR) (29). As a proinflammatory mediator, PAFR can activate immune cells involved in asthma exacerbation, inducing eosinophil chemotaxis and mucus hypersecretion (29).

SA colonization has been confirmed as one of the risk factors for asthma progression. A significant association exists between SA colonization and the incidence of asthma in children (32). The pathogenic mechanisms of SA in asthma involve the induction of inflammatory responses in airway epithelial cells, thereby exacerbating inflammation and affecting the function of respiratory epithelial cells and eosinophils (33). Patients with asthma exhibit specific IgE antibodies against SA-secreted enterotoxins (e.g., SEA and SEB), indicating an allergic response (34, 35). Another superantigen produced by SA is toxic shock syndrome toxin-1 (TSST-1). Both SEB and TSST-1 can induce Th2-type inflammatory pathways, promote the secretion of inflammatory factors (such as IL-13), activate Th2 cells to stimulate the production of IgE by plasma cells, increase eosinophil infiltration in the airways, activate ILC2s, and induce airway hyperresponsiveness (AHR) (36).

The core mechanisms by which bacterial pathogens induce or exacerbate asthma are identical. However, owing to the distinct pathogenic characteristics and colonization sites of different bacteria, these mechanisms also exhibit specific characteristics. Bacteria directly damage the airway epithelial barrier and secrete toxins that induce the secretion of IL-33, thymic stromal lymphopoietin (TSLP), and other cytokines from airway epithelial cells, thereby promoting the activation of Th2 cells and ILC2s, stimulating the secretion of inflammatory factors (such as IL-13), activating B cells to produce IgE, and increasing eosinophil infiltration and mucus hypersecretion in the airways. Chronic inflammation induced by persistent bacterial infection leads to airway remodeling, characterized by thickening and metaplasia of the surface epithelium, hyperplasia of goblet cells, and hypertrophy of smooth muscle, resulting in irreversible structural changes in the airways and the induction of AHR (37).

### 3.2 Pediatric asthma with viral infection

Respiratory viral infections are the leading cause of hospitalization for infants worldwide and the second leading cause of infant mortality (38). Numerous viruses contribute to respiratory infections, with RSV and RV being the most prevalent. Other common pathogens include *influenza viruses*, *parainfluenza viruses*, and *coronaviruses* (39). RSV is an enveloped, nonsegmented, negative-strand RNA virus belonging to the Pneumoviridae family (40). Multiple studies have confirmed the association between RSV and asthma pathogenesis (41). Following RSV infection, hospitalized infants develop structural and functional sequelae, leading to persistent pulmonary dysfunction characterized by chronic airflow limitation and adverse airway remodeling (42). Sigurs et al. (43) reported that children with RSV bronchiolitis in early life have an increased risk of developing asthma. A retrospective cohort study conducted by Maarten et al. (44) demonstrated that infant RSV infection is associated with increased hospitalization rates for recurrent wheezing and asthma, particularly during the preschool years. Children with a history of RSV lower respiratory tract infection (LRTI) are 2–12 times more likely to develop asthma than those without such a history (45). RSV infection is associated with susceptibility to asthma in children through several mechanisms. First, RSV infection downregulates the expression of tight junction proteins such as Zo-1, increasing epithelial permeability and disrupting the airway epithelial barrier (46). Second, following RSV infection, airway epithelial cells release innate cytokines that activate Th2 immune responses through interactions with dendritic cells (DCs), T cells, and ILC2s, recruiting eosinophils and increasing mucus secretion. Additionally, RSV infection impairs DC function through the expression of stimulatory molecules such as CD80 and CD86, affects CD4^+^ T-cell function, inhibits IFN-γ production, suppresses Th1 immune responses, and reduces the antiviral capacity of the body (47). Moreover, RSV infection leads to epigenetic changes in mucosal and systemic immune cells, thus causing airway inflammation and pulmonary vascular remodeling and creating an immune microenvironment that promotes asthma (48).

RVs are single-stranded RNA viruses without an envelope and belong to the genus RV of the family Picornaviridae (49). RV infection is also associated with the development of asthma. Compared with patients infected with RSV, individuals with wheezing illnesses caused by RV infection have much more severe symptoms and a significantly greater risk of developing asthma (50). In mouse models, early-life RV infection leads to long-term alterations in airway physiology (51). During RV infection, activation of the innate immune mechanism drives allergic responses, contributing to the development and exacerbation of asthma. Compared with healthy individuals, individuals with experimental RV infection exhibit a significant decline in lung function and exacerbated lower respiratory tract symptoms (52). RV infection in neonatal mice induces airway inflammation, mucus metaplasia, and hyperresponsiveness. In RV-infected young mice, the numbers of immune cells, including neutrophils, macrophages, lymphocytes, eosinophils, and ILC2s, increase, accompanied by increased expression of IL-13, IL-25, IL-33, and TSLP (53). RV infection can enhance AHR, which is closely associated with the induction of proinflammatory cytokines such as IL-6 (54). Compared with RSV infection, RV infection tends to induce higher levels of airway inflammation, which is characterized primarily by the infiltration of eosinophils. Persistent inflammatory responses and excessive activation of immune cells stimulate fibroblast activation and smooth muscle cell proliferation, accompanied by increased mucus production and bronchoconstriction, leading to AHR and promoting the development of airway remodeling (55, 56).

Influenza viruses belong to the Orthomyxoviridae family. During peak influenza seasons, the infection rate among infants with asthma may reach as high as 20% (57). Children with asthma are particularly susceptible to influenza virus infection. An American study that tracked participants’ symptoms and detected nasal swab samples demonstrated that between 2009 and 2010, children with asthma were twice as likely to be infected with H1N1 influenza as children without asthma were (58). Influenza viruses specifically target ciliated epithelial cells in the airways, disrupting the protective airway barrier. Damaged epithelial cells secrete alarmins such as TSLP, IL-33, and IL-25, which stimulate ILC2 activation and the secretion of effector factors, thereby inducing AHR. Furthermore, influenza viruses can inhibit regulatory T cells, promote Th2 cell activation, induce allergen-specific IgE production, and activate Th17 cells, with IL-17 mediating neutrophil recruitment (59, 60). In healthy children, infection with the influenza virus primarily activates Th1 cells, which secrete IFN-γ to clear the virus and exert antiviral functions. In contrast, children with asthma exhibit unbalanced Th1 and Th2 immunity, characterized by a propensity toward the Th2 pathway (59, 60).

Severe acute respiratory syndrome coronavirus 2 (SARS-CoV-2) is the primary pathogen responsible for COVID-19. It is an enveloped, single-stranded RNA virus belonging to the genus *Betacoronavirus*. A retrospective cohort study conducted in the United States involving 46,900 children aged 5–17 years revealed that the risk of SARS-CoV-2 infection in children with asthma was similar to that in children without asthma (61). A meta-analysis by Wimwipa et al. (62) revealed that asthma was not a risk factor for hospitalization or intensive care admission in children with SARS-CoV-2 infection. This might be attributed to the fact that parents of children with asthma may have had greater concerns about asthma control during the respiratory disease pandemic, thus improving their children's medication adherence and further enhancing asthma management (63). SARS-CoV-2 infection invades airway epithelial cells via ACE2, leading to the release of alarmins from these cells. Consequently, antiviral Th1 responses are suppressed, and the Th2 and Th17 pathways become activated. Moreover, SARS-CoV-2 can directly impact airway neuroregulation, causing bronchoconstriction, hypersecretion of mucus, and induction of AHR. Following infection, children are more prone to developing a state of long-term immune immaturity, which manifests as recurrent wheezing, new-onset asthma, and deterioration in the control of preexisting asthma (64, 65).

### 3.3 Pediatric bronchial asthma and *Mycoplasma pneumoniae* infection

Mpn, a prokaryotic pathogen intermediate between viruses and bacteria, lacks both cell walls and membranes. It is characterized by its ability to adhere to and invade respiratory epithelial cells, triggering oxidative stress responses and causing mucosal damage in the respiratory tract (66). Jiao et al. (67) reported that compared with children without asthma, children with asthma presented higher serum IgE levels, eosinophil counts, and IgM-positive rates. Furthermore, Mpn infection was strongly associated with elevated IgE concentrations, increased eosinophil counts, and a greater proportion of IgM-positive patients. A meta-analysis of 22 studies examined the relationship between Mpn infection and asthma in children. The findings revealed that the odds ratios (ORs) of children with recent Mpn infections for asthma were 3- and 7-fold greater than those for healthy children. These findings demonstrate a significant association between Mpn infection and the development of asthma in children (68). Biscardi et al. (69) reported that 50% of patients with acute asthma exacerbations had acute Mpn infection. In a follow-up study involving 50 children, 5 (10%) with Mpn-related respiratory disease developed clinical asthma symptoms (70). Pathologically, Mpn infection damages epithelial cells through CARDS toxins, leading to increased permeability of epithelial cells, inducing a Th2-type immune response, and promoting increased production of IL-4 and IL-5, which in turn facilitate AHR and eosinophilic inflammation. Mpn infection increases Th17 cells and IL-17 levels, inhibits Treg function, and exacerbates airway inflammation and tissue damage. Chronic Mpn infection stimulates fibroblast proliferation, resulting in thickening of the airway wall, luminal narrowing, and airway remodeling (68, 71, 72).

Cpn, a member of the Chlamydiaceae family, is a type of obligate intracellular parasitic prokaryotic microorganism that occupies an intermediate position between bacteria and viruses (36). In a 10-year observational study, researchers tracked microbiological and clinical data from 10 newly diagnosed cases of Cpn. Among 9 patients with acute bronchitis and wheezing, 4 experienced symptom improvement without treatment, while 5 progressed to chronic asthma (73). Horvat et al. (74) demonstrated through neonatal mouse models that Chlamydia infection exacerbates the development of hallmark asthma features in ovalbumin-induced allergic airway disease models. Cpn infection can trigger Th2 immune responses and increase the secretion of inflammatory factors, including eosinophilia and neutrophil infiltration in the airways, an increase in the number of mucus-secreting cells and AHR, and permanent alterations in lung structure and function, thereby promoting airway remodeling (75).

## 4 Effects of obesity on pediatric asthma and lung infection

The global prevalence of childhood obesity is approximately 8.5%, and 254 million children are projected to be affected by obesity by 2,030 (76). When the body's caloric intake exceeds its expenditure, excessive energy is converted into fat stored in the body, leading to systemic fat accumulation and obesity. Adipose tissue plays a crucial role in energy storage and metabolism throughout the body (77). In addition to secreting hormones that regulate various physiological processes, adipose tissue also serves as a hub for inflammatory responses (78). Adipose tissues contain macrophages, which can be polarized into two main phenotypes, namely, “M1” and “M2” macrophages. “M1” macrophages exhibit proinflammatory characteristics and release proinflammatory factors such as tumour necrosis factor alpha (TNF-α), IL-1β, and IL-6, whereas “M2” macrophages demonstrate anti-inflammatory properties that help regulate and suppress inflammatory processes. In obese individuals, “M1” macrophages predominate in adipose tissue, contributing to the formation of a proinflammatory microenvironment (79). For example, obesity appears to reduce airflow in the upper respiratory tract while inducing certain proinflammatory changes (80).

### 4.1 Clinical association between pediatric obesity and asthma

The incidence and severity of pediatric asthma are closely linked to obesity. A meta-analysis revealed that compared with children of normal weight, overweight children have a 20% greater incidence of asthma, whereas children with obesity face a 40% greater risk of asthma (81). Children with a high body mass index (BMI) and obesity are significantly more likely to have asthma (82). In children with obesity and asthma, increased secretion of proinflammatory molecules (such as IL-6 and TNF-α) and leptin is accompanied by decreased adiponectin levels (83). In animal experiments, mice in the leptin intervention group presented increased AHR, along with elevated levels of vascular IgE and IL-6 production in the airway (84). Studies have indicated that compared with children with asthma and normal weight, those with asthma who are overweight or obese are more prone to pulmonary function abnormalities (85, 86). For example, children with obesity and asthma present increased forced vital capacity (FVC) and forced expiratory volume in one second (FEV1) but a decreased FEV1/FVC ratio, which suggests impaired pulmonary function in this population (85, 87). Children with obesity have a higher morbidity rate, poorer quality of life, and more severe symptoms of asthma. In addition, children with obesity and asthma are more likely to develop resistance to corticosteroids and bronchodilators, the medications used for asthma treatment. A study conducted by Forno et al. (86) analyzed 1,041 children aged 5–12 years with mild-to-moderate persistent asthma and reported that when corticosteroids were used for treatment, the improvement in children with normal weight was significantly greater than that in children with overweight or obesity.

### 4.2 Clinical association between obesity and pulmonary infection

Obesity is often associated with altered immune responses, increased susceptibility to acute respiratory infections, and increased severity (88–90). For example, a study of 61 hospitalized children under 18 years of age in Taiwan with H1N1 infection revealed that obesity was significantly associated with increased ICU demand and mortality (88). During both the H1N1 pandemic and nonpandemic influenza seasons, obesity increases the risk of hospitalization due to influenza A virus (IAV) infection (89, 90). During lower respiratory tract infection, obese children have significantly increased morbidity and mortality rates, along with increased risks of secondary infections (91, 92). In animal experiments, obese mice with IAV infection exhibit greater pulmonary hyperventilation than lean mice do, accompanied by pulmonary edema and exacerbated oxidative stress (93). Docherty et al. (94) conducted a classification study of approximately 20,000 hospitalized SARS-CoV-2-positive patients across 208 UK hospitals and reported that approximately 10% of the patients were obese. Furthermore, a Mexican cohort study involving 51,633 SARS-CoV-2-positive patients revealed that compared with those patients of normal weight, patients with obesity had significantly higher mortality rates and required more intensive care (95). Soyak et al. (25) enrolled 981 children with asthma, of whom 189 had concurrent COVID-19. Compared with the pre-infection status, forced expiratory flow (FEF) levels of 25%–75% were significantly lower in these children (*P* < 0.001), with obesity identified as an independent risk factor for this change.

However, several studies have also suggested that obesity is not an independent risk factor for asthma exacerbations or pulmonary infections. Test et al. (96) conducted a cohort analysis of 716 children (82 children with overweight and 138 children with obesity) and revealed that among hospitalized children with asthma or pneumonia, obesity was not associated with poor health-related quality of life (HRQOL) scores at admission, 2–6 weeks post-discharge, hospital length, or 30-day follow-up. A retrospective study by Neyer et al. (97) at a major pediatric hospital revealed no statistically significant association between severe obesity and influenza complications (*P* = 0.61), indicating that severe childhood obesity is not an independent risk factor for influenza and its complications.

### 4.3 Mechanisms of obesity in asthma and pulmonary infection

Some studies have explored the potential link between obesity and the risk of pulmonary infection or related mortality in obese mouse models. The results demonstrated that compared with lean mice, ob/ob obese mice infected with pneumonia caused by *Streptococcus pneumoniae* or *Klebsiella* presented a marked increase in mortality or death risk, along with a weaker ability to clear bacteria from the lungs and blood (98, 99). In diet-induced obese mice and leptin-deficient obese mice, elevated levels of proinflammatory cytokines, including MCP-1, TNF-α, IL-6, and TNF-α, have been observed (100). These aberrant changes exert systemic effects and impair the pulmonary immune microenvironment. For children with obesity, resident or recruited immune cells within adipose tissue, such as macrophages, DCs, NK cells, B cells, and T cells, undergo dysregulation, which may lead to abnormal immune function in the context of prolonged childhood obesity.

Several potential mechanisms by which obesity influences asthma and pulmonary infections have been revealed: 1) Chronic inflammation: obesity leads to increased infiltration of macrophages into adipose tissue and enhanced M1 polarization, which promotes the proliferation of Th1 cells and increases the levels of IL-6, IL-10, IFN-γ, and TNF. Dysregulated secretion of adipokines in adipose tissue, characterized by elevated leptin levels and reduced adiponectin levels, promotes AHR. Prolonged obesity induces systemic chronic inflammation, creating a proinflammatory microenvironment in which proinflammatory cytokines directly activate airway immune cells, amplify inflammatory responses, increase mucus secretion, and increase AHR. 2) Epithelial barrier dysfunction: Persistent inflammatory responses damage epithelial cell tight junctions, compromising barrier integrity and facilitating pathogen invasion, thereby exacerbating infections and asthma. 3) Alterations in pulmonary function and airway mechanics: Excessive accumulation of thoracic and abdominal fat exerts pressure on the lungs and reduces lung volume, with the most pronounced changes being decreases in functional residual capacity and expiratory reserve volume. Reduced radial traction of the lung parenchyma on the airways may lead to airway collapse and increased airway resistance. Additionally, obesity may alter airway smooth muscle function. Concurrently, systemic inflammation induced by obesity may promote airway epithelial fibrosis and thickening of the airway wall, resulting in luminal narrowing, exacerbating AHR, and facilitating airway remodeling. Notably, children represent a critical period for pulmonary development (25, 85, 87, 101).

## 5 The impact of type 1 diabetes mellitus on pediatric asthma and pulmonary infections

Type 1 diabetes mellitus (T1DM) is an autoimmune disorder that selectively destroys pancreatic beta cells, resulting in absolute insulin deficiency. Patients with T1DM require lifelong daily insulin injections (102, 103). Globally, the incidence of T1DM is increasing at a rate of approximately 3%–5% per year, with a more pronounced trend in children younger than 5 years (104). Approximately 50%–60% of patients with T1DM are diagnosed before the age of 15 years. In Western countries, T1DM accounts for more than 90% of pediatric and adolescent diabetes cases (105).

In recent years, the incidence rates of both global asthma and T1DM have been on the rise (106). Both are common chronic diseases in children. The exact mechanisms underlying their interaction remain unclear. The key mechanisms of T1DM may involve Th1 cell-mediated processes. Studies have indicated that elevated Th1 cells in patients with T1DM may correspond to a reduction in Th2 cells, suggesting a potential negative correlation between asthma (Th2-associated) and T1DM (Th1-associated) (107–109). Research has shown that the prevalence of asthma is similar between patients with T1DM and patients without T1DM. Patients with TIDM exhibit a higher incidence of asthma (110–113). Furthermore, a study from Denmark revealed that children with asthma have a 20%–30% increased risk of developing T1DM (114). Additionally, children cosuffering from both asthma and T1DM often face greater challenges in glycemic control and typically require higher insulin doses to achieve therapeutic efficacy (115).

Studies indicate that patients with T1DM are more susceptible to pneumococcal pneumonia, not only facing a higher risk of infection but also exhibiting more severe clinical manifestations and poorer prognoses (116). Furthermore, during influenza seasons, compared with patients without TIDM, patients with T1DM experience more severe disease progression and additional complications. Compared with wild-type mice, diabetic mice are more prone to infection with *influenza A virus* (IAV) and display greater symptom severity and mortality rates, which can be ameliorated by insulin treatment (117). During the 2009 H1N1 influenza pandemic, compared with patients without TIDM, patients with T1DM had a threefold increased risk of hospitalization and a fourfold increased risk of intensive care unit admission following viral infection (118). In a large retrospective study conducted in the United States, children with T1DM admitted to the hospital for COVID-19 exhibited more severe disease symptoms than children without T1DM did (119).

Children with T1DM exhibit immune system dysregulation and certain immunodeficiencies. Elevated blood glucose levels reduce immune system activity and induce alterations in tissues, skin, and blood flow, all of which contribute to an increased risk of infections and asthma. The potential mechanisms through which T1DM may influence asthma and pulmonary infections involve multiple pathways. 1) Impaired innate immunity: Hyperglycemia impairs macrophage function, downregulates key mediators of the host's innate humoral and cell-mediated immune responses to various pathogens, reduces the synthesis of proinflammatory cytokines, and increases the virulence of multiple pathogens (120). 2) T1DM is characterized by a Th1-dominant profile, whereas asthma is Th2 predominant. Defective Treg function during childhood may lead to an imbalance and the coexistence of these profiles, exacerbating asthma symptoms (121). 3) Disruption of respiratory mucosal barriers: Hyperglycemia may promote epithelial apoptosis, increase mucus density, and diminish pathogen clearance capacity, facilitating pathogen invasion. Hyperglycemia-induced reactive oxygen species (ROS) generation activates the nuclear factor kappaB (NF-κB) pathway, leading to increased release of proinflammatory cytokines (e.g., IL-6 and TNF-α) and the formation of a proinflammatory microenvironment, resulting in rapid progression of inflammation following infection (122). 4) Airway remodeling: Hyperglycemia may cause pulmonary microvascular damage, increased permeability, and aggravated airway edema (123). Concurrently, abnormal calcium homeostasis in airway smooth muscle enhances contractility and significantly increases airway hyperresponsiveness. The accumulation of advanced glycated end products activates the advanced glycation end-products (ARGE) pathway, potentially accelerating airway remodeling through the promotion of airway smooth muscle proliferation and collagen deposition, thereby exacerbating irreversible asthma damage (124). T1DM increases the risk and severity of pulmonary infections in children and influences airway function via potential mechanisms; conversely, pulmonary infections can further exacerbate asthma pathology.

## 6 Effects of metabolic alterations in the gut microbiota on asthma and pulmonary infections

### 6.1 The impact of altered gut microbiota metabolism on pediatric asthma and pulmonary infections

A variety of microorganisms that coexist symbiotically with the host reside within the human intestinal tract and play crucial roles in maintaining physical health and disease development. Arrieta et al. (125) analyzed the gut microbiota of 319 infant participants and reported that children with gut microbiota dysbiosis have a greater risk of developing asthma. Researchers have reported that the relative abundance of beneficial bacteria in the gut microbiota of children diagnosed with asthma is reduced, leading to decreased levels of short-chain fatty acids. Supplementation with these beneficial bacteria can alleviate symptoms. Chen et al. (126) analyzed the differences in the gut microbiota between 167 children with bronchial asthma and 167 healthy children. Children with asthma exhibited lower levels of the beneficial bacterial taxa *Bifidobacterium* and *Lactobacillus* in their feces, while the enrichment of conditionally pathogenic bacteria such as *Escherichia coli* and *Enterococcus* was greater. With increasing asthma severity, the levels of *Bifidobacterium* and *Lactobacillus* decrease sequentially, whereas the enrichment of *Escherichia coli* and *Enterococcus* increases. Pulmonary microbiota have been confirmed to exist in healthy individuals, albeit with significantly lower biomass than that of the gut microbiota. When pulmonary diseases such as asthma occur, the pulmonary microbiota undergoes a certain degree of ecological imbalance (127). Wang et al. (128) investigated the sputum microbiome of patients with asthma and reported that a reduction in gram-negative bacterial abundance was associated with asthma, whereas Spn enrichment increased in the respiratory microbiome of patients with asthma.

The gut and lungs interact through the gut–lung axis, which is essential for maintaining immune homeostasis. The mesenteric lymphatic system serves as a critical pathway between the lungs and the gut. Intact bacteria, their fragments, or metabolic products can traverse the intestinal barrier via this system, enter the systemic circulation, and modulate pulmonary immune responses (129). In a healthy state, the gut microbiota maintains the balance of Th1/Th2 immune responses by regulating DC function, thereby suppressing asthma-associated inflammation. Conversely, gut dysbiosis disrupts this balance, driving excessive activation of Th2 immune responses and leading to elevated IL-4 levels and eosinophil infiltration, which promote asthma inflammation and pulmonary infections (130).

Dysbiosis of the gut microbiota is often accompanied by alterations in secondary metabolites [such as short-chain fatty acids (SCFAs), bile acids (Bas), and tryptophan metabolites], which are closely associated with human health. By activating the G protein-coupled receptors 41 and 43 (GPR41/43) and Peroxisome proliferator-activated receptor gamma (PPARγ) ligands, SCFAs increase the bactericidal capacity of macrophages and DCs and suppress Th2 immune responses to reduce allergic reactions in the airway. Additionally, SCFAs inhibit the release of proinflammatory factors in the airways to protect the epithelial barrier, thereby reducing pulmonary infections and alleviating asthma (131, 132). Gut microbiota dysbiosis leads to insufficient SCFAs, damage to the intestinal epithelial barrier, and increased intestinal permeability. Moreover, altered gut microbiota weakens pulmonary immune defense and increases pulmonary susceptibility (131, 132). BAs protect against airway inflammation through multiple mechanisms, including via the pulmonary FXR receptor, which attenuates eosinophilic airway inflammation and reduces allergen-induced airway inflammatory responses, mucus metaplasia, and AHR (132). Tryptophan metabolites can activate the aryl hydrocarbon receptor, promoting the differentiation of DCs, Th17 cells, and Treg cells and inhibiting ILC2 function to influence the homeostasis of intestinal innate lymphoid cells (133, 134).

**Figure 2:**
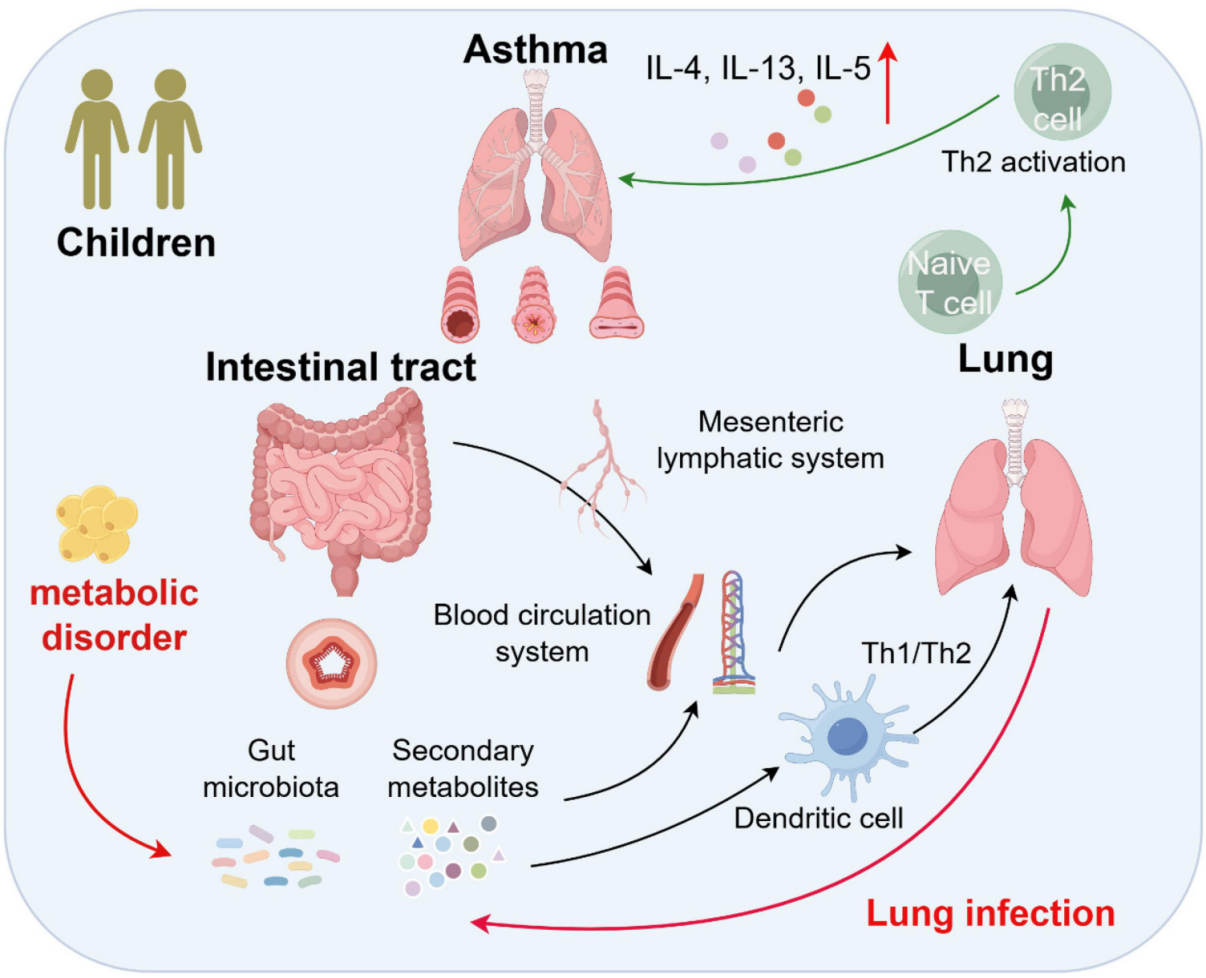
Effects of metabolic alterations in the gut microbiota on childhood asthma and pulmonary infections. The mesenteric lymphatic system serves as a vital pathway between the lungs and the gut. Intact bacteria and metabolites (e.g., short-chain fatty acids, SCFAs) can cross the intestinal barrier through this system, enter systemic circulation, and regulate pulmonary immune responses. The gut microbiota maintains the Th1/Th2 balance by modulating dendritic cell (DC) function, thereby suppressing asthma-associated inflammation. Conversely, gut microbiota dysbiosis disrupts this balance, driving excessive Th2 immune responses that increase inflammatory cytokine levels and induce eosinophil infiltration, ultimately promoting asthma progression.

These findings demonstrate a significant correlation between early gut microbiota changes and pediatric asthma development (Figure 2). Although studies have investigated the mechanism of this association, the specific mechanisms remain unclear, making it a key focus for future research.

### 6.2 The impact of MetS on pediatric asthma and pulmonary infections

The diagnostic criteria for MetS include abdominal obesity (increased waist circumference), hypertension, hypertriglyceridemia, low high-density cholesterol, and elevated fasting blood glucose levels. The estimated overall prevalence of MetS in children and adolescents in 2020 was 2.8% and 4.8%, respectively (135). MetS may mediate changes in innate immune responses through systemic inflammation, IR, and altered lipid metabolism, involving the ILC2 and Th17 pathways, and plays a key role in adipose tissue homeostasis and asthma (136). MetS can induce systemic chronic inflammation, reduce host defense capabilities, and precipitate asthma exacerbation (137). By upregulating proinflammatory factors such as TNF-α and IL-6, MetS contributes to airway epithelial damage and increases the risk of respiratory tract infections. Oxidized lipids resulting from dyslipidemia can induce the release of inflammatory cytokines, upregulate the expression of endothelial cell adhesion molecules, and potentially increase eosinophilic inflammation, such as mucus hypersecretion, AHR, and subepithelial fibrosis (138).

The primary characteristics of metabolic dysfunction-associated steatotic liver disease (MASLD) include abnormal liver function, lipid metabolic dysregulation, impaired IR, lipotoxicity, and enhanced inflammatory responses (139). The prevalence of MASLD in children varies by region, with an overall prevalence of 52.49% in obese individuals and 7.40% in the general population. It is projected that this proportion will reach 30.7% by 2,040 (140). MASLD may serve as a risk factor for altered pulmonary function in children. Compared with children with overweight/obesity without MASLD, children aged 8–14 years with MASLD exhibit significantly reduced lung function. IL-1β and IR may act as key risk factors contributing to airway dysfunction in children with MASLD. Furthermore, the enhanced inflammatory response in MASLD may induce chronic pulmonary inflammation and increase susceptibility to pulmonary infections through the release of large amounts of inflammatory cytokines and adipokines via the “liver–lung axis,” thus participating in airway remodeling processes. However, whether MASLD affects childhood asthma and the specific mechanisms involved require further experimental validation (141).

## 7 Conclusion and perspective

To summarize, this review discusses the mutual interactions between metabolic abnormalities, pulmonary infections, and bronchial asthma. We suggest that metabolic disorders may induce susceptibility to pulmonary infections and exacerbate asthma through mechanisms such as influencing immune cells, inflammatory responses, and airway barrier function. Pulmonary infections serve as a primary risk factor for asthma exacerbation and disease worsening in children. Metabolic disorders such as obesity, T1DM, MASLD, and MetS impair immune function, amplify inflammatory responses, and contribute to the formation of a vicious cycle characterized by “metabolic dysregulation–immune dysfunction–infection susceptibility–asthma exacerbation”.

Early metabolic disorders in children deserve increased attention. These disorders not only increase the risk of pediatric asthma but also affect asthma treatment. They reduce the sensitivity of affected children to therapeutic drugs, meaning that higher doses are required to control the condition, which in turn increases the incidence of adverse drug reactions and side effects. If early interventions can be implemented in children—such as regular physical activity and a balanced diet—to maintain a healthy gut microbiota and prevent metabolic disorders, they will not only reduce the risk of asthma exacerbations but also increase children's resistance to infections.

Weight gain is both a common clinical outcome of metabolic disturbances and a typical manifestation of metabolic disorders, such as obesity and MetS. Therefore, weight management can alleviate metabolic disturbances by reducing body weight. Primary weight reduction measures include dietary interventions, mainly by controlling the consumption of low-calorie balanced diets; restricting the intake of carbohydrates and saturated fats; and increasing the proportions of dietary fiber, high-quality protein, and unsaturated fatty acids, thereby helping prevent and mitigate the severity of asthma (142). In an experiment comparing the Mediterranean diet and the low-fat diet, both diets were able to reduce hepatic steatosis and improve insulin sensitivity in children with MASLD within 12 weeks (143). Exercise intervention involves developing personalized aerobic exercise programs, such as brisk walking, swimming, and cycling, on the basis of the child's age, asthma control status, pulmonary function, and other physical data to reduce sedentary behavior and screen time. By increasing energy expenditure, body fat percentage can be decreased, and asthma can be alleviated (144). An 18-month randomized controlled weight loss trial (diet management and exercise intervention) conducted on 87 children with overweight/obesity with asthma revealed clinically relevant improvements in weight, pulmonary function, and asthma characteristics in both the intervention and control groups, with more pronounced effects in the intervention group regarding forced vital capacity, asthma control, and quality of life (145).

Pharmacological adjuvant intervention may be utilized under the guidance of specialist evaluations for children with severe metabolic disorders (including diabetes mellitus, severe obesity, and MetS) who have not responded adequately to lifestyle interventions (diet and exercise), such as metformin and glucagon-like peptide-1 (GLP-1) receptor agonists, to assist in weight reduction and metabolic improvement. Animal models have demonstrated that metformin can prevent vagally induced AHR and may exert anti-inflammatory effects within the airways. Additionally, metformin may have a role in ameliorating asthma severity (146). Treatment with GLP-1 receptor agonists may inhibit eosinophilic infiltration induced by airway allergens (147). Statins, which possess anti-inflammatory and immunomodulatory properties, may contribute to asthma control. Animal studies have confirmed that pravastatin inhibits allergic airway infiltration and AHR by suppressing Th2 and Th17-related signaling pathways, reducing leptin expression, and attenuating downstream p38 MAPK signaling. However, this effect was not observed in lean asthma mouse models (148). Dysbiosis of the gut microbiota is also a risk factor in children with comorbid metabolic abnormalities and asthma. Probiotic intervention has been reported to improve the severity of MASLD and BMI. In murine models, probiotic intervention has been shown to mitigate the severity of increased airway reactivity in mice with obesity and asthma (149).

This review presents certain limitations, including the limited sample size of current relevant clinical studies, a focus on animal experiments in mechanism research, and insufficient evidence regarding long-term prognosis in the pediatric population. Future research should conduct large-scale prospective cohort studies that integrate metabolic regulation, microbiota intervention, and anti-inflammatory and anti-infective strategies to provide new evidence for the precise prevention and treatment of childhood asthma.
